# Effects of a Mediterranean Diet-Based Program on Cognitive Decline: Non-Blinded Non-Randomized Controlled Trial of the CESPORT Program

**DOI:** 10.3390/nu18071073

**Published:** 2026-03-27

**Authors:** Juan Carlos Checa Olmos, Montserrat Monserrat Hernández, Ángeles Arjona Garrido, Jose Antonio Salinas, Manuel Díaz-Pérez

**Affiliations:** 1 Sociology Area, Department of Geography, History and Humanities, University of Almería, 04120 Almería, Spain; jcheca@ual.es; 2 Anthropology Area, Department of Geography, History and Humanities, University of Almería, 04120 Almería, Spain; arjona@ual.es; 3 Department of Engineering, University of Almería, CIAMBITAL Research Center, Agrifood Campus of International Excellence (CeiA3), 04120 Almería, Spain; jsalinas@ual.es (J.A.S.); madiaz@ual.es (M.D.-P.)

**Keywords:** Mediterranean diet, diet therapy, CESPORT program, cognitive decline, BDNF, Cognifit^®^

## Abstract

**Background**: Age-related cognitive deccline is a significant health issue in Spain, especially among adults over 60 years of age. Addressing this involves establishing intervention guidelines and identifying early diagnostic biomarkers. **Objective:** To evaluate changes in urine of Brain-Derived Neurotrophic Factor, concentration and cognitive performance after the implementation of the multicomponent CESPORT program (incorporating a Mediterranean Diet, nutritional education, and continuous support). **Methods**: This controlled trial included 76 older adults, divided into an experimental group (n = 58; mean age 66.9 years; 75.9% female) that participated in the CESPORT program, and a control group (n = 18; mean age 68.8 years; 72.2% female). Cognitive performance was assessed using the Mini-Mental State Examination (MMSE) and the Cognifit^®^ battery. Urinary BDNF concentrations were quantified via ELISA. **Results**: After adjusting for baseline scores via ANCOVA, the experimental group demonstrated significantly higher post-intervention outcomes compared to the control group (*p* < 0.001). Substantial improvements with medium-to-large effect sizes were observed in global cognition, reasoning, attention, coordination and perception. Furthermore, urinary BDNF levels were significantly elevated in the experimental group. Positive correlations were found between Brain-Derived Neurotrophic Factor concentrations and cognitive performance in multiple domains (*p* < 0.05), particularly regarding global status and reasoning. **Conclusions**: The multicomponent CESPORT intervention demonstrates a potential protective effect against age-related cognitive decline. Furthermore, urinary BDNF emerges as a promising, non-invasive early biomarker for cognitive health. Further research is warranted to validate these findings.

## 1. Introduction

The increase in life expectancy has changed aging patterns. According to the World Health Organization, the proportion of the global population over 60 years of age will nearly double from 12% to 22% between 2015 and 2050 [1]. Specialists often identify the age of 60 as a critical threshold for the onset of age-related cognitive decline [2], which typically affects orientation, language, perception, visuospatial skills, and attention [3]. This decline, an inherent aspect of physiological aging, is distinct from Alzheimer’s disease and other dementias [4]; crucially, it can be mitigated or even partially reversed through targeted physical and mental activities, nutritional interventions, and stress management [5,6]. At the physiological level, this deterioration is driven by mechanisms such as amyloid deposition, oxidative stress, synaptic degeneration, cholinergic dysfunction and neuroinflammation [7]. Specifically, oxidative stress reduces the expression of TrkB, the primary receptor for Brain-Derived Neurotrophic Factor (BDNF)—a protein crucial for neurogenesis, neuroprotection, and neuronal survival [8].

BDNF has been extensively studied as a key biomarker of cognitive function, with higher circulating levels consistently associated with enhance learning, memory, and synaptic plasticity in both animal and human studies [9,10]. Mechanistically, BDNF binds to TrkB receptors, activating intracellular signaling cascades—including PI3K/Akt, MAPK/ERK, and PLC-γ pathways—that promote dendritic growth, synaptic plasticity, and resistance to oxidative stress [9,10]. Nutritional interventions, particularly high adherence to the Mediterranean Diet (MD), have proven effective in upregulating circulating BDNF levels, thereby providing a molecular rationale for the cognitive improvements observed in recent clinical studies [10,11,12,13]. The MD is characterized by a high intake of vegetables, legumes, fish, and extra virgin olive oil, alongside a low intake of saturated fats. It is rich in bioactive compounds, such as polyphenols and vitamins A, B, C, and E, which exert potent antioxidant effects that further support BDNF expression and overall neuronal health [8,12,13]. Furthermore, the MD offers antidiabetic, lipid-lowering, and antihypertensive benefits [6,8], while the broader Mediterranean lifestyle promotes outdoor activities that favorably influence endogenous vitamin D synthesis and overall status [14].

The impact of cognitive decline is frequently exacerbated by common age-related comorbidities, such as hypertension, diabetes, and malnutrition [15], underscoring the urgent need for early diagnosis and comprehensive lifestyle interventions.

In Spain, Clinical Practice Guideline for Alzheimer’s disease and other dementias strongly recommend early screening for suspected cognitive impairment [16]. Tools such as the Mini-Mental State Examination (MMSE) [16,17] and the Mini Cognitive Exam (MEC) [18] are widely employed for objective monitoring, although their accuracy can be influenced by the patient’s educational attainment. Other validated screening instruments (e.g., SPMSQ, MIS, T7M, TdR, Eurotest, Fototest, and T@M) [19] and computerized cognitive training platforms (e.g., Cogmed^®^, Lumosity^®^, or NeuronUP^®^) are also utilized. While the Spanish national health system implements various non-pharmacological interventions—such as cognitive stimulation workshops and memory training—these approaches are often resource-intensive and frequently overlook foundational self-care lifestyle factors, particularly diet and physical activity [15].

Based on this mechanistic and empirical evidence, we postulate that a comprehensive nutritional and lifestyle intervention could successfully modulate BDNF levels and support neuroplasticity, thereby improving cognitive outcomes. To address this, we developed the CESPORT program (Cognition, Exercise, and Social support in older adults through the PoRTuguese and Spanish Mediterranean Diet), a multidisciplinary intervention designed to enhance MD adherence through direct food provision and nutritional education, seamlessly integrated with physical activity and social support. Therefore, this study aims to evaluate the changes in urinary BDNF concentrations and cognitive test performance following the implementation of this multicomponent CESPORT program. We hypothesize that participation in the program will yield significant improvements in both global cognitive function and urinary BDNF levels, and that these two outcomes will be positively correlated.

## 2. Materials and Methods

### 2.1. Study Desing

The experimental design is based on a non-blinded, non-randomized controlled trial. Although a 1:1 allocation ratio was initially considered, the final distribution resulted in an unequal ratio (approximately 3:1) to observe the influence of the CESPORT program on the cognitive health of the participants. This decision was primarily driven by ethical considerations and resource optimization within the framework of the CESPORT program, aiming to maximize the number of participants benefiting from the food provision and nutritional intervention. Additionally, the final sample size in each group was influenced by participant availability and the logistical constraints of delivering weekly food baskets during the study period. To account for this imbalance, all statistical analyses were performed using robust models (ANCOVA) to ensure that the unequal group sizes did not compromise the validity of the results. In this study, the TREND guidelines were followed for the method and results.

### 2.2. Participants

The sample was obtained from individuals over 60 years enrolled at the senior university in a region of southern Spain. All participants were informed about the study and gave consent to participate.

Eligibility criteria included being older than 60 years, no diagnosed neurodegenerative or cardiovascular diseases, and not taking medications that could affect cognition (acetylcholine blockers, anxiolytics, hypnotics, corticosteroids, statins, chemotherapy, or chronic pain treatments).

The sample size was determined a priori using STATA-13 software. The calculation was based on detecting at least a medium effect size (e.g., Cohen’s *d* = 0.50) for the primary outcomes (cognitive improvement and BDNF variations). This expected effect size was derived from previous similar studies evaluating the impact of the Mediterranean Diet on neurotrophic factors and cognitive function, which commonly report small-to-moderate effect sizes [20]. Assuming a two-tailed hypothesis, an alpha error probability (α) of 0.05, and a statistical power (1 − β) of 80%, the minimum required sample size was calculated. An unequal allocation ratio was intentionally and prospectively established, driven primarily by ethical considerations and resource optimization, to maximize the number of participants benefiting from the CESPORT program’s direct food provision. Finally, the total recruited sample was adjusted to account for potential dropouts during the 4-month follow-up period and the number of significance tests performed.

The study included two parallel groups: the experimental group participated in the CESPORT program, while the control group did not receive any intervention.

Although no previous studies have specifically examined urinary BDNF after a Mediterranean Diet-based program, earlier cross-sectional research in Spain suggested cognitive benefits. In that study, adherence to the Mediterranean Diet increased MMSE scores by 0.16 points in the experimental group, compared to a −0.26 decrease in the control group. This corresponds to a 6% difference in mean scores, highlighting the potential impact of the intervention and supporting the proposed sample size and expected outcomes. Formula applied: n_1_ = (σ2 1 + σ2 2 K) × [(zα + zβ)2/(μ1 − μ2)] [21], where n_1_ is the required sample size for the intervention group; σ_1_^2^ is the variance of the outcome variable in group 1; σ_2_^2^ is the variance of the outcome variable in group 2; k is the ratio between the sample sizes of the groups; zα is the critical value of the standard normal distribution associated with the significance level; zβ is the value associated with the Type II error; μ_1_ is the expected mean outcome for group 1; and μ_2_ is the expected mean outcome for group 2.

### 2.3. Procedure

An open, non-blinded intervention was applied for 4 months within the CESPORT program (from February 2023, to May 2024) to students enrolled at senior university of a region in southern Spain. After obtaining approval from the Ethics Committee on Human Research from the researchers’ university (UALBIO2023/048), an informational meeting took place to provide information to the volunteers about the development of the project. A detailed explanation was provided about the aims and purposes of the project.

After this, those who were interested signed an informed consent form. Due to the non-randomized nature of the study, participants were not assigned through randomization blocks or codes. Instead, the allocation into the control and experimental groups was conducted via a convenience sampling approach. The research team assigned participants based on their personal availability, their willingness to commit to the 4-month program, and the logistical capacity of the CESPORT program to deliver the weekly food baskets. This pragmatic approach ensured the viability of the nutritional intervention while resulting in the naturally unequal group distribution. Due to the logistical nature of the CESPORT program and real-world constraints, formal allocation concealment mechanisms were not employed. Consistent with the open-label design, neither the participants nor the staff delivering the nutritional education were blinded to the intervention. Furthermore, the outcome assessor (the neuropsychologist responsible for administering the Cognifit^®^-Cognifit España SL, Madrid, Spain- and MMSE evaluations) was also aware of the participants’ group assignments. While this non-blinded design introduces a potential risk of observer bias, all cognitive assessments were strictly conducted following standardized computerized protocols (Cognifit^®^) to ensure objective data collection and minimize subjective interpretation. Additionally, the use of ANCOVA models adjusted for baseline scores further mitigates the impact of potential systematic biases in the final results.

During the process, the experimental group took part in four information sessions about the adequate cooking and use of food in the MD. Also, they were provided with a recipe book to cook the food received weekly, and they were also part of a WhatsApp group for them to write down any questions they may have about the process. Specifically, the CESPORT intervention lasted for 4 months. Weekly, participants received baskets of fruits and vegetables provided by Coprohnijar, whose nutrients have been identified in recent research as “epigenetic protectors” [8,12,13]. The vegetable basket was composed of 7 tomatoes, 2 green peppers, 2 red peppers, 2 yellow peppers, 4 zucchinis, 4 cucumbers, and 2 eggplants. The micronutrient content of the basket described in Table 1 was analyzed by the external laboratory Labcolor^®^ (Labcolor Coexphal, Almería, Spain), accredited according to ISO 17025 [22]. At the start of the intervention, four workshops were conducted to instruct participants on how to prepare and consume these foods to maximize nutrient absorption. Each week, participants received their fresh produce basket to support adherence to the dietary program. Specifically, the products provided were irrigated with rain water from rain water collection installations located in each greenhouse, water from a desalinating plant, and underground water sources from existing nearby wells. The composition of these waters, as well as the nutrients provided by the food in each basket and irrigated with these waters (without a cost for the participants), are shown in Table 1. Conversely, the control group did not receive any food provision, nutritional education, or specific dietary guidelines. They were instructed to maintain their usual, unrestricted (ad libitum) dietary habits throughout the 4-month study period. The actual dietary intake and variability for both the experimental and control groups were objectively assessed and compared using the validated FFQ at baseline and post-intervention. 

### 2.4. Instrument

Dietary data were collected at baseline (pre-intervention) during a face-to-face interview with a trained dietitian using a validated 137-item Food Frequency Questionnaire (FFQ) widely utilized and validated for the Spanish population (specifically, the one used in the PREDIMED study) [13] and was administered again at the end of the 4-month intervention to assess changes in dietary adherence (MDS) and confirm the successful implementation of the nutritional program. This instrument evaluates the frequency of consumption of different food groups over the previous month to accurately estimate daily dietary intake. Additionally, daily physical activity and exercise habits were assessed using the short form of the International Physical Activity Questionnaire (IPAQ) at both time points, which categorizes the frequency, duration, and intensity of weekly physical activities. The Mediterranean Diet Score (MDS) index is calculated by assigning a value of 0 or 1 to nine components of daily intake. Following the original Mediterranean Diet Score (MDS) methodology developed by Trichopoulou et al. [23], and the specific guidelines adapted by Giménez [12] and Hutchins-Wiese [24], the sex-specific median intake of the study sample was used as the cutoff point for each dietary element. A value of 1 is assigned if the consumption of foods that are considered protectors is equal to or higher than the calculated median intake of these foods, and if the consumption of damaging foods is lower to the median intake. Thus, the MDS is obtained by assigning a score of 0/1 if the daily intake of 9 components, in which the following are assessed: monounsaturated fatty acids/saturated fatty acids ratio (MUFA/SFA); a high consumption of vegetables, legumes, fruits and nuts, cereals and fish; moderate consumption of alcohol, milk, and milk products; and low consumption of meat and meat derivatives. In the case of the present work, items related to the type of milk-derived products were added (non-fat, low-fat, or whole), types of cereals (whole grain or white), type of fish (white or blue), and type of meat (red or white), to be able to obtain more detailed information.

Just as in the study by Giménez in 2001 [12], the mean intake was calculated for each element in the diet, differentiating, for each of the components, what type they were. The participants received a positive point if their intake was higher than the mean for components understood to be “protectors” (vegetables, legumes, fruits, nuts, cereals, and fish), and zero if their intake was higher than the mean of the sample, for components considered “non-protectors” (composed of saturated fats and simple sugars). For the MUFA/SFA ratio, a score of 1 was considered above the mean, and zero in the opposite case.

In general, the sum of all the components can vary from 0 to 9, where “0” is a lack of adherence to the Mediterranean Diet, and “9” is the maximum level of adherence (considered good starting at 7 points) [23].

The values of weight, BMI, and percentage of body fat were estimated with a TANITA BC-545N^®^ bioimpedance (TANITA EUROPE B.V., Amsterdam, The Netherlands) scale. Height was measured with a Soehnle 5003 ^®^ electronic (Soehnle Professional GmbH & Co. KG, Backnang, Germany) height rod.

Evaluation of cognitive function: An experienced neuropsychologist performed the cognitive examinations. The instruments used were the Mini-Mental State Examination (MMSE) [16,17] to measure the overall cognitive function, and to determine the cognitive domains (reasoning, memory, attention, coordination, and perception), the battery of diagnostic questions of the online program Cognifit^®^ was utilized. With regard to the first (MMSE), it consists of 30 questions with items grouped into 5 sections that verify orientation, short-term memory, attention, calculation, deferred memory, and language and construction (score up to 30 points), with a sensitivity of 89.8%, specificity of 75.1%, and α = 0.920 for the Spanish population [17]. With respect to the second (Cognifit^®^), it lasted approximately 30–40 min and was performed through its online application. At the end of the assessment, a complete report of the results was obtained with the neurocognitive profile of the user. The cognitive profile of the neuropsychological report for individuals older than 60 and of Spanish nationality had a high reliability, consistency, and stability (α = 0.900 test–retest with values close to 1) [25].

Laboratory determinations: To find if there were changes at the physiological level, the BDNF values were measured in urine (peripheral) at the start and end of the intervention. Regarding the pre-analytical conditions for urinary BDNF assessment, urine samples were collected under standardized parameters to minimize variability. Participants provided a mid-stream urine sample early in the morning, between 8:00 and 9:00 AM, following an overnight fast of approximately 8 to 12 h. To avoid extreme fluctuations in urine concentration, participants were instructed to maintain their standard hydration habits but avoid excessive fluid intake on the morning of the collection. Samples were promptly centrifuged, aliquoted, and stored at −80 °C until the ELISA analysis was performed. The sample analysis was performed at an independent laboratory (Biogenox, Almería, Spain) with a BDNF-ELISA (Enzyme-Linked Immunosorbent Assay) kit (IBL International, Hamburg, Germany, ref. E-EL-H0010-96T), with a sensitivity of 18.75 pg/mL, a minimum detection limit of 31.25 pg/mL, CV intra-assay < 10% and CV inter-assay < 12%, and a high specificity, due to the lack of cross-reactions with any of the cytokines tested. The target protein was quantified in the sample, the mean absorbance was plotted against the concentration of the protein, and a curve fitted to the standard result was created. Then, the absorbance of the samples was interpolated to the curve to calculate the concentration. This was all performed with the data analysis software ELISA curve Expert (v1.324, Daniel Hyams, Madison, AL, USA). This measurement kit was used given its recognition by the scientific community to be used in urine with this type of protein specifically [26,27]. Concerning the handling of urine dilution, BDNF concentrations were reported as absolute values (pg/mL) and were not normalized to urinary creatinine levels. As this study aimed to explore urinary BDNF as a rapid, accessible, and non-invasive biomarker in a community-dwelling senior population, the raw values were utilized in accordance with the standard instructions of the commercial immunoassay kit. We acknowledge the absence of creatinine normalization as a methodological limitation regarding hydration status control, which has been addressed in the limitations section.

### 2.5. Data Analysis

The data analysis was performed using the SPSS software (v28.0, IBM Corp. Armonk, NY, USA). Prior to the main analyses, the assumptions of normality and homogeneity of variance were verified using the Shapiro–Wilk and Levene’s tests, respectively. For the ANCOVA models, the assumption of homogeneity of regression slopes was also verified.

To evaluate the effectiveness of the multicomponent CESPORT program, an Analysis of Covariance (ANCOVA) was utilized for each cognitive outcome (MMSE and Cognifit^®^ domains: memory, reasoning, perception, coordination, and attention) and urinary BDNF levels. This approach was chosen to assess differences between the experimental and control groups over time while statistically controlling for initial baseline values. For each model, the post-intervention score was entered as the dependent variable, group assignment (experimental vs. control) as the fixed factor, and the baseline (pre-intervention) score as the covariate. Effect sizes for the main effects were reported using partial eta squared (η_p_^2^), and 95% confidence intervals were calculated for the adjusted mean differences.

For the analysis of correlations between all the cognitive variables and BDNF, Spearman’s Rho was used, with a two-tailed level of significance.

Finally, Odds-ratio (OR) tests were performed to observe if there was an influence of the intervention program between the experimental group (EG) and control group (CG) on the probability of obtaining BDNF values in urine below the 10th percentile (<26 µg/mL).

## 3. Results

**Figure 1:**
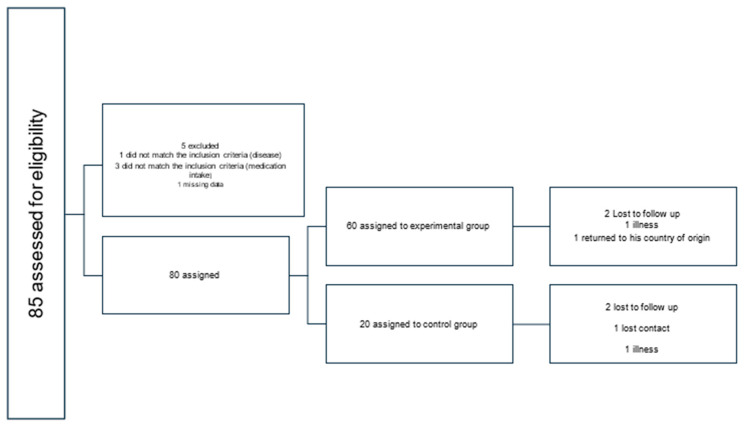
Flowchart of subject progress through phases of the non-randomized study. Source: Own elaboration.

The population that completed all the tests consisted of 76 individuals, 58 belonging to the experimental group (M_age_ = 66.93, SD = 3.97) (24% men and 75.9% women) and 18 to the control group (M_age_ = 68.77; SD = 3.42) (27.8% men and 72.2% women) (Figure 1).

The descriptive baseline data (Table 2) show that the control group (CG) had a lower Body Mass Index (BMI) and body fat percentage (%) compared to the experimental group (EG) and also engaged in less daily physical activity. Conversely, EG participants spent fewer hours per day on sedentary activities. With respect to initial adherence to the Mediterranean Diet (MDS), both groups exhibited similar baseline mean scores.

To confirm the successful implementation of the nutritional program, these parameters were evaluated descriptively at the end of the 4-month study. As expected, the EG demonstrated a notable increase in their mean MDS (7.54 ± 1.70), reflecting high adherence, whereas the CG maintained a lower score (5.47 ± 1.98). To provide a detailed perspective on these dietary shifts, the pre- and post-intervention consumption data for the nine specific food components comprising the MDS for both groups are detailed in Appendix A (Table A1). Regarding anthropometric changes, the EG descriptively showed a slight reduction in BMI (Post-intervention: 26.96 ± 4.71 kg/m^2^) and body fat (post-intervention: 34.15 ± 7.85%) compared to their initial values. In contrast, the CG remained relatively stable in both BMI (Post-intervention: 25.22 ± 2.38 kg/m^2^) and body fat (post-intervention: 30.40 ± 6.76%).

**Figure 2:**
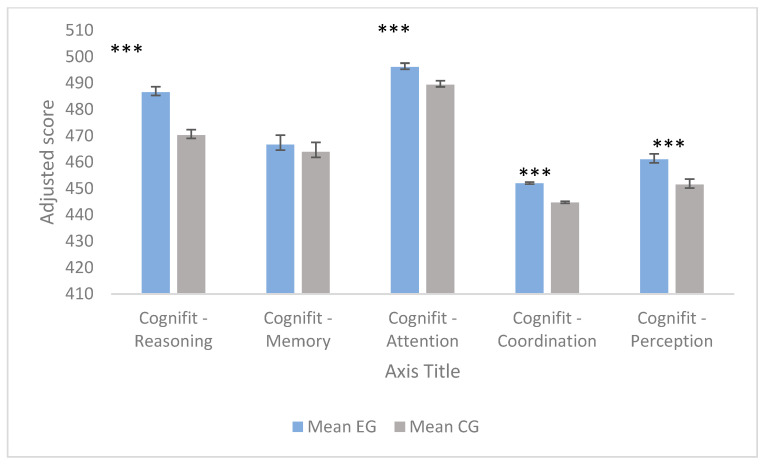
Estimated marginal means for the Cognifit^®^ cognitive domains following the 4-month intervention. Data are adjusted for baseline scores using ANCOVA models to rigorously account for initial inter-individual variability. Error bars represent the Standard Error (SE). Asterisks indicate statistically significant differences between the experimental group (n = 58) and the control group (n = 18) (*** *p* < 0.001). Note: While the experimental group shows visually prominent raw increases across Cognifit dimensions (including memory), the ANCOVA models adjusting for baseline differences determined that the memory domain change was not statistically significant between groups. Source: Own elaboration.

**Figure 3:**
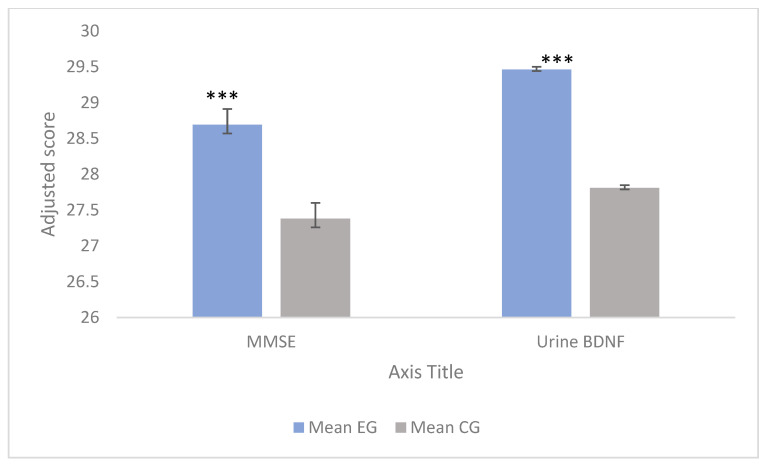
Estimated marginal means for the Mini-Mental State Examination (MMSE) and urinary BDNF levels following the 4-month intervention. Data are adjusted for baseline scores using ANCOVA models. Error bars represent the Standard Error (SE). Asterisks indicate statistically significant differences between the experimental group (n = 58) and the control group (n = 18) (*** *p* < 0.001). Note: While the experimental group shows visually prominent raw increases across Cognifit dimensions (including memory), the ANCOVA models adjusting for baseline differences determined that the memory domain change was not statistically significant between groups. Source: Own elaboration.

The results of the Analysis of Covariance (ANCOVA) used to evaluate the effectiveness of the multicomponent CESPORT program on cognitive variables and BDNF levels are visually represented in Figure 2 and Figure 3. By adjusting for baseline (pre-intervention) scores as covariates, we were able to isolate the true effect of the intervention. To avoid data redundancy in the main text, the comprehensive numerical data for these analyses are detailed in Appendix A (Table A2 and Table A3).

The analysis revealed that the experimental group (EG) demonstrated significantly better adjusted post-intervention outcomes compared to the control group (CG) in almost all evaluated domains. Regarding the Cognifit^®^ dimensions (Figure 2), the EG achieved substantial and statistically significant higher adjusted means compared to the CG in reasoning (EG: M = 486.50, SE = 2.12 vs. CG: M = 470.22, SE = 1.19; *p* < 0.001, η_p_^2^ = 0.352), attention (EG: M = 496.08, SE = 1.49 vs. CG: M = 489.39, SE = 0.84; *p* < 0.001, η_p_^2^ = 0.156), coordination (EG: M = 452.00, SE = 0.49 vs. CG: M = 444.68, SE = 0.28; *p* < 0.001, η_p_^2^ = 0.675), and perception (EG: M = 461.02, SE = 2.17 vs. CG: M = 451.46, SE = 1.23; *p* < 0.001, η_p_^2^ = 0.148). Conversely, the robust ANCOVA confirmed no significant between-group differences for memory (EG: M = 466.66, SE = 3.55 vs. CG: M = 463.93, SE = 2.07; *p* = 0.509, η_p_^2^ = 0.005), indicating that the 4-month intervention did not produce differential improvements in this specific domain.

Furthermore, concerning overall cognitive status and neuroplasticity markers (Figure 3), the adjusted analysis confirmed highly significant improvements for the EG in both global cognition measured by the MMSE (*p* < 0.001; η_p_^2^ = 0.147) and urinary BDNF concentrations (*p* < 0.001; η_p_^2^ = 0.931). These results suggest that the changes in these variables are directly linked to the multicomponent intervention.

The results of the correlation analyses revealed distinct patterns before and after the intervention. Prior to the program, significant positive correlations (*p* ≤ 0.001) were observed among all Cognifit^®^ dimensions—reasoning, memory, attention, coordination, and perception—indicating strong positive interrelationships among the different cognitive domains. Furthermore, a significant association emerged between baseline BDNF and MMSE scores (r = 0.225; *p* ≤ 0.005), as well as between MMSE and both memory (r = 0.162; *p* ≤ 0.05) and coordination (r = 0.191; *p* ≤ 0.05). After the intervention, these associations became stronger and more widespread. Post-intervention BDNF showed positive and statistically significant correlations with MMSE (r = 0.319; *p* ≤ 0.001), reasoning (r = 0.233; *p* ≤ 0.001), coordination (r = 0.203; *p* ≤ 0.05), and perception (r = 0.228; *p* ≤ 0.001), suggesting that higher levels of BDNF were linked to better cognitive performance across several domains. Additionally, the relationships among the Cognifit^®^ dimensions remained significant (*p* ≤ 0.005), particularly between reasoning and coordination (r = 0.567), and between memory and perception (r = 0.580), reflecting the multidimensional nature of the cognitive state observed in participants. All detailed correlation coefficients and their confidence intervals can be found in Appendix A (Table A4 and Table A5).

The Odds-ratio (OR) analysis showed a significant association (*p* = 0.029). The estimated OR of 0.233 (95% CI: 0.057–0.944) indicates a strong protective effect of the intervention. Specifically, the probability of obtaining riskily low BDNF values (below the 10th percentile) was significantly higher in the CG (22.2% within the group) compared to the EG (5.2% within the group). Consequently, this protective OR demonstrates that participants in the experimental group had a significantly higher probability of maintaining optimal or elevated BDNF levels compared to the control group. 

## 4. Discussion

The main objective of this study was to evaluate whether cognitive changes following the implementation of the multicomponent CESPORT program, which encompasses a Mediterranean Diet (MD) intervention, nutritional education, and continuous participant support. Designed as a non-randomized controlled trial, the 4-month CESPORT program aimed to increase MD adherence through food provision, educational workshops, and a digital community. The intervention yielded significant improvements in both cognitive tests scores and physiological neuroplasticity markers, specifically BDNF levels. These findings are particularly novel because few clinical trials have evaluated the impact of a multicomponent MD-based program on distinct cognitive domains, and even fewer have demonstrated a direct relationship between urinary BDNF and cognitive performance.

Our results expand upon previous research conducted in Spain regarding the benefits of the MD [8,12,13], further reinforcing its role as a protective factor against cognitive decline [8]. These neuroprotective effects are largely attributed to the hih polyphenol content and monounsaturated fatty acids derived from extra virgin olive oil and nuts [8,13,27]. Furthermore, by employing a pre- and post-intervention design, our study strengthens the evidence for temporal associations between dietary changes and cognitive health.

With respect to global cognition, MMSE scores improved significantly in the experimental group (EG). Previous epidemiological studies and systematic reviews have linked serum concentrations of specific antioxidants and nutrients with enhanced cognitive performance, including higher MMSE scores, across cross-sectional, prospective, and case–control designs. However, these associations are often evaluated based on isolated nutrients rather than a holistic dietary pattern [28]. In this context, the CESPORT program provides novel evidence supporting the preventive efficacy of multicomponent, MD-based interventions.

Similary, for the Cognifit^®^ battery, although the EG presented with lower baseline scores, the ANCOVA revealed significant post-intervention improvements in reasoning, attention, coordination, and perception compared to the control group. The observed correlations between MMSE and Cognifit^®^ further validate the utility of this digital tool. Notably, our robust analysis found no significant between-group differences in the memory domain. While the literature has reported nutrient–cognition associations specifically related to memory [29], the lack of significant improvement in our study may be attributed to the relatively short 4-month intervention period. This duration might be insufficient to induce the structural or functional neuroplastic changes required in memory-specific neural networks, suggesting that longer interventions may be necessary to detect subtle effects on memory.

There are also other possible mechanisms of brain protection through the MD, such as the increase in neurotrophic factors related to neurotransmission. The exact biological mechanisms underlying these cognitive benefits are likely multifactorial. The MD is highly rich in polyphenols (abundant in extra virgin olive oil, nuts, and vegetables) and omega-3 polyunsaturated fatty acids (mainly from fish). These bioactive compounds have been shown to exert potent antioxidant and anti-inflammatory effects, effectively reducing neuroinflammation. Furthermore, both omega-3 fatty acids and dietary polyphenols are known to upregulate the expression of Brain-Derived Neurotrophic Factor (BDNF), which promotes neurogenesis, synaptic plasticity, and neuronal survival.

Regarding this neurotrophin, a sub-study [30] observed higher plasma BDNF levels in patients with depression who followed a MD, although these differences were not statistically significant. In our study, which employed a shorter intervention period, significant differences were found between the experimental and control groups in the post-intervention analysis of urinary BDNF (but not in the pre-intervention analysis). This BDNF-mediated pathway provides a strong biological rationale for the significant cognitive improvements observed in our experimental group. Nevertheless, both groups improved their scores relative to baseline. These results may be explained by participation in the University for Seniors program and the practice of physical activity, present in both experimental and control groups, which has also been shown to influence BDNF production [31].

This study also presents a novel methodological approach by analyzing urinary BDNF, a less invasive alternative to serum analysis. Consistent with the growing body of literature highlighting the critical utility of peripheral biochemical biomarkers in evaluating cognitive decline and dementia in older adults [32,33], we observed that lower urinary BDNF levels correlated with worse cognitive scores, supporting its potential for rapid, non-invasive early detection in primary care.

Furthermore, our findings offer critical insights into how to effectively modulate these neuroplasticity markers. While the literature has extensively explored single-component interventions, results have often been inconsistent. For instance, isolated supplementation with specific nutrients (such as vitamin E or B vitamins) or herbal extracts like Ginkgo biloba has generally failed to demonstrate robust and long-lasting efficacy in preventing cognitive impairment [27]. Similarly, standalone physical activity programs often yield mixed results on global cognitive function and neurotrophic factors like BDNF when not accompanied by nutritional improvements [8]. In this context, our findings are particularly relevant. They emphasize that a synergistic, multicomponent approach—like the CESPORT program, which integrates the complex matrix of bioactive compounds from the Mediterranean Diet with lifestyle support—is a significantly more effective strategy for enhancing BDNF levels and preserving cognitive health than isolated treatments.

Several limitations must be acknowledged. First, there were baseline differences in Cognifit^®^ scores between groups, although our use of ANCOVA statistically adjusted for these initial disparities. Second, the study features a non-blinded design where group allocation was performed by the researchers, and crucially, the neuropsychologist evaluating the cognitive outcomes was not blinded to group assignment, which may have introduced observer bias. Third, the sample sizes were unequal, and the control group was relatively small (58 vs 18). Fourth, the repeated administration of cognitive tests over a short 4-month follow-up period raises the possibility of practice effects (learning effects), which could partially account for the intra-group improvements observed. Fifth, regarding the urinary BDNF methodology, our protocol did not include creatinine normalization to adjust for urine dilution and hydration status, a pre-analytical factor that should be controlled in future research. Finally, the observed improvements may result not only from MD adherence but also from the overall CESPORT program (motivation, belonging, dietary advice).

## 5. Conclusions

In conclusion, the findings of this study demonstrate that participation in the multicomponent CESPORT program yields significant improvements across multiple cognitive domains—including those assessed by the Cognifit^®^ battery and the MMSE—as well as in physiological neuroplasticity markers such as urinary BDNF. Our data strongly reinforces the clinical relevance of urinary BDNF, which exhibits a significant positive correlation with cognitive performance in areas such as perception, reasoning, and coordination. Notably, the association between lower urinary BDNF concentrations and poorer cognitive scores, coupled with the higher probability of the control group falling below the 10th percentile for BDNF levels, highlights the neurocognitive vulnerability of older adults who do not engage in structured multicomponent interventions.

These results suggest that, rather than relying on single-domain treatments, public health strategies should prioritize integrated programs that combine adherence to the Mediterranean Diet with physical activity and social engagement. We strongly recommend the implementation of such multidisciplinary approaches in primary care settings and community senior centers as a robust, non-invasive strategy to prevent early cognitive decline. Furthermore, our findings advocate for the use of urinary BDNF as a highly promising, accessible, and non-invasive biomarker for monitoring cognitive health, thereby facilitating the early identification of older adults at risk of neurocognitive impairment.

## Figures and Tables

**Table 1:** Description of the nutrients found in the water and the foods provided to the experimental group for weekly consumption.

Parameter/Nutrient	Intervention Composition (Mean ± SD)	EFSA Daily Recommendations *
Part A: Irrigation Water		
Carbonates (mg/L)	0.00 ± 0.00	--
Bicarbonates (mg/L)	259.29 ± 130.60	--
Chlorides (mg/L)	617.01 ± 410.20	--
Sulfates (mg/L)	384.09 ± 299.71	--
Nitrates (mg/L)	26.94 ± 38.75	--
Calcium (mg/L)	87.15 ± 61.11	--
Sodium (mg/L)	120.48 ± 80.44	--
Potassium (mg/L)	394.78 ± 275.06	--
Zinc (mg/L)	21.19 ± 20.44	--
Iron (mg/L)	0.23 ± 0.39	--
Manganese (mg/L)	0.07 ± 0.05	--
Copper (mg/L)	0.03 ± 0.02	--
Boron (mg/L)	0.08 ± 0.18	--
Part B: Food Consumed (CESPORT Basket)	Weekly Basket (Mean ± SD)	Daily target
Magnesium (mg)	371.60 ± 44.33	350
Iron (mg)	16.90 ± 2.60	11
Vitamin B3 (mg)	17.90 ± 3.16	11.2
Vitamin C (mg)	1207.80 ± 20.03	110
Vitamin E (mg)	31.60 ± 5.44	12
Dietary fiber (g)	46.87 ± 4.96	25
Potassium (mg)	6710.50 ± 87.44	3400
Vitamin B1 (mg)	1.47 ± 0.26	0.7
Vitamin B6 (mg)	3.16 ± 0.42	1.7
Vitamin A (µg)	1451.80 ± 32.88	750
Monounsaturated fatty acids (mg)	83.08 ± 2.72	20% of total fat
Calcium (mg)	738.20 ± 1.64	950
Folic acid (µg)	1082.40 ± 20.83	330

Note: * The general recommendations for dietary consumption by program participants were obtained from the recommendations of the European Food Safety Authority [11]. Source: Own elaboration.

**Table 2:** Baseline characteristics of participants.

Variable	EG (n = 58)	CG (n = 18)	Total (n = 76)	*p*-Value
Age (years)	66.93 ± 3.97	68.77 ± 3.42	67.85 ± 3.78	0.12
BMI (kg/m^2^)	27.99 ± 4.72	25.34 ± 2.36	26.67 ± 3.77	0.08
Body fat (%)	35.77 ± 7.68	29.89 ± 6.83	32.83 ± 7.46	0.04
	Women 37.66 ± 7.43 Men 29.83 ± 4.75	Women 30.40 ± 7.70 Men 28.56 ± 2.96	Women 36.01 ± 8.06 Men 29.50 ± 4.35	
Hours of sleep	9.68 ± 1.81	9.05 ± 1.05	9.36 ± 1.52	0.15
Mediterranean Diet Score (MDS)	5.54 ± 1.40	5.38 ± 2.03	5.46 ± 1.73	0.72
MUFA_SFA ratio	1.28 ± 0.53	1.15 ± 0.30	1.21 ± 0.44	0.30
DH physical activity	0.85 ± 0.84	1.13 ± 0.41	0.99 ± 0.68	0.21
DH sedentary	4.37 ± 1.18	5.27 ± 1.48	4.82 ± 1.38	0.05

Note: EG = experimental group; CG = control group; DH = daily hours; BMI = Body Mass Index; MUFA = Monounsaturated Fatty Acids; SFA = Saturated Fatty Acids. Source: Own elaboration.

## Data Availability

The datasets generated and analyzed during the current study are available in the Zenodo repository, at the following DOI: https://doi.org/10.5281/zenodo.17256268.

## Abbreviations

The following abbreviations are used in this manuscript:

ANCOVA	Analysis of Covariance
BDNF	Brain-Derived Neurotrophic Factor
BMI	Body Mass Index
CG	Control Group
EG	Experimental Group
FFQ	Food Frequency Questionnaire
IPAQ	International Physical Activity Questionnaire
MD	Mediterranean Diet
MDS	Mediterranean Diet Score
MMSE	Mini-Mental State Examination
MUFA	Monounsaturated Fatty Acids
PUFA	Polyunsaturated Fatty Acids
SFA	Saturated Fatty Acids

## Appendix A

**Table A1:** Mean daily intake of the 9 Mediterranean Diet Score (MDS) components before and after the 4-month intervention.

MDS Component (Units)	Control Group (Pre)	Control Group (Post)	Experimental Group (Pre)	Experimental Group (Post)
Protective components				
Vegetables (g/day)	180.5 ± 45.2	175.2 ± 50.1	185.3 ± 40.5	320.4 ± 60.2
Legumes (g/day)	15.2 ± 8.4	16.0 ± 9.1	14.8 ± 7.9	35.5 ± 12.4
Fruits and nuts (g/day)	210.3 ± 60.1	205.4 ± 65.3	215.6 ± 55.4	380.2 ± 70.1
Cereals (g/day)	150.4 ± 40.2	155.1 ± 38.4	148.5 ± 45.1	190.5 ± 40.8
Fish and seafood (g/day)	45.6 ± 20.1	48.2 ± 22.5	42.4 ± 18.5	85.6 ± 25.4
MUFA/SFA ratio				
Ratio (lipids)	1.15 ± 0.30	1.16 ± 0.41	1.28 ± 0.53	1.81 ± 0.51
Damaging components				
Meat and derivatives (g/day)	160.5 ± 50.2	165.4 ± 48.6	155.4 ± 55.2	90.2 ± 30.5
Dairy products (g/day)	250.4 ± 80.5	245.6 ± 75.4	260.5 ± 85.2	180.4 ± 50.6
Moderate components				
Alcohol (g/day) *	12.5 ± 8.4	13.0 ± 9.1	14.2 ± 7.5	10.5 ± 5.2

* Data are presented as Mean ± Standard Deviation. MUFA: Monounsaturated Fatty Acids; SFA: Saturated Fatty Acids. Alcohol consumption is considered optimal when intake is moderate (e.g., 10–50 g/day for men, 5–25 g/day for women).

**Table A2:** Analysis of Covariance (ANCOVA) for cognitive outcomes and BDNF adjusting for baseline scores.

Variable	Control Group Adjusted Mean (SE)	Experimental Group Adjusted Mean (SE)	F	*p*-Value	η_p_^2^
MMSE	27.38 (0.22)	28.69 (0.12)	25.70	<0.001	0.147
Cognifit-Reasoning	470.22 (1.19)	486.50 (2.12)	44.53	<0.001	0.352
Cognifit-Memory	463.93 (2.07)	466.66 (3.55)	0.44	0.509	0.005
Cognifit-Attention	489.39 (0.84)	496.08 (1.49)	15.21	<0.001	0.156
Cognifit-Coordination	444.68 (0.28)	452.00 (0.49)	163.87	<0.001	0.675
Cognifit-Perception	451.46 (1.23)	461.02 (2.17)	14.60	<0.001	0.148
Urine BDNF	27.810 (0.042)	29.460 (0.024)	1158.43	<0.001	0.931

Note: BDNF = Brain-Derived Neurotrophic Factor; MMSE = Mini-Mental State Examination. Source: Own elaboration.

**Table A3:** Descriptive statistics (raw scores) and ANCOVA pairwise comparisons (adjusted differences, confidence intervals, and standardized effect sizes) for cognitive outcomes and urinary BDNF.

Variable	Group	Pre-Intervention	Post-Intervention	Adjusted Mean Difference	95% CI of the Difference	*p*-Value	Effect Size
MMSE	Exp.	27.74 (1.67)	28.93 (1.59)	1.311	[0.800, 1.822]	<0.001	0.147
	Cont.	26.44 (2.00)	26.61 (2.61)				
Reasoning	Exp.	420.91 (129.74)	465.65 (129.62)	16.280	[11.427, 21.133]	<0.001	0.352
	Cont.	489.27 (107.41)	478.16 (111.76)				
Memory	Exp.	414.70 (113.55)	467.12 (107.54)	2.733	[−5.451, 10.917]	0.509	0.005
	Cont.	459.16 (125.47)	466.66 (127.78)				
Attention	Exp.	438.48 (130.92)	488.94 (126.49)	6.692	[3.279, 10.105]	<0.001	0.156
	Cont.	485.33 (92.43)	492.77 (96.92)				
Coordination	Exp.	393.00 (149.73)	446.41 (137.23)	7.318	[6.180, 8.456]	<0.001	0.675
	Cont.	454.89 (91.08)	452.00 (85.48)				
Perception	Exp.	429.34 (121.40)	460.98 (124.81)	9.569	[4.590, 14.548]	<0.001	0.148
	Cont.	462.66 (107.76)	460.88 (104.74)				
UrineBDNF	Exp.	28.44 (2.11)	29.24 (2.56)	1.651	[1.554, 1.747]	<0.001	0.931
	Cont.	27.06 (4.13)	27.80 (2.41)				

Note: ‘Raw Mean (SD)’ represents the unadjusted descriptive statistics. ‘Adjusted Mean Difference’ represents the estimated marginal mean difference between the experimental and control groups, controlling for baseline (pre-intervention) scores. CI = Confidence Interval η_p_^2^ = Partial Eta Squared (standardized effect size). Source: Own elaboration.

**Table A4:** Results of the pre-intervention correlation analysis.

	BDNF	Reasoning	Memory	Attention	Coordination	Perception	MMSE
BDNF (urine)	1	0.129	0.132	0.017	0.136	0.143	0.225 **
Reasoning (Cognifit)		1	0.567 **	0.433 **	0.602 **	0.538 **	0.039
Memory (Cognifit)			1	0.463 **	0.519 **	0.606 **	0.162 *
Attention (Cognifit)				1	0.451 **	0.484 **	0.099
Coordination (Cognifit)					1	0.557 **	0.191 *
Perception (Cognifit)						1	0.238 **
MMSE							1

Note: * Level of significance ≤ 0.05; ** Level of significance ≤ 0.001. BDNF = Brain-Derived Neurotrophic Factor. MMSE = Mini-Mental State Examination. Source: Own elaboration.

**Table A5:** Results of the post-intervention correlation analysis.

	BDNF	Reasoning	Memory	Attention	Coordination	Perception	MMSE
BDNF (urine)	1	0.233 **	0.149	0.052	0.203 *	0.228 **	0.319 **
Reasoning (Cognifit)		1	0.510 **	0.333 **	0.567 **	0.449 **	0.108
Memory (Cognifit)			1	0.391 *	0.452 **	0.580 **	0.163 *
Attention (Cognifit)				1	0.448 **	0.402 **	0.130
Coordination (Cognifit)					1	0.432 **	0.155
Perception (Cognifit)						1	0.189 *
MMSE							1

Note: * Level of significance ≤ 0.05; ** Level of significance ≤ 0.001. BDNF = Brain-Derived Neurotrophic Factor. MMSE = Mini-Mental State Examination. Source: Own elaboration.
